# A Modular System for Detection, Tracking and Analysis of Human Faces in Thermal Infrared Recordings †

**DOI:** 10.3390/s19194135

**Published:** 2019-09-24

**Authors:** Marcin Kopaczka, Lukas Breuer, Justus Schock, Dorit Merhof

**Affiliations:** Institute of Imaging and Computer Vision, RWTH Aachen University, 52062 Aachen, Germany

**Keywords:** thermal infrared imaging, image processing, face detection, face analysis

## Abstract

We present a system that utilizes a range of image processing algorithms to allow fully automated thermal face analysis under both laboratory and real-world conditions. We implement methods for face detection, facial landmark detection, face frontalization and analysis, combining all of these into a fully automated workflow. The system is fully modular and allows implementing own additional algorithms for improved performance or specialized tasks. Our suggested pipeline contains a histogtam of oriented gradients support vector machine (HOG-SVM) based face detector and different landmark detecion methods implemented using feature-based active appearance models, deep alignment networks and a deep shape regression network. Face frontalization is achieved by utilizing piecewise affine transformations. For the final analysis, we present an emotion recognition system that utilizes HOG features and a random forest classifier and a respiratory rate analysis module that computes average temperatures from an automatically detected region of interest. Results show that our combined system achieves a performance which is comparable to current stand-alone state-of-the-art methods for thermal face and landmark datection and a classification accuracy of 65.75% for four basic emotions.

## 1. Introduction

The thermal signature of objects yields information that cannot be obtained in the visual spectrum. In recent years, advances in thermal image sensor design have led to a number of commercially available imaging systems that allow analyzing thermal images with resonable effort. Since then, thermal or long-wave infrared (LWIR) imaging has gained increasing attention as imaging modality for analysis of both human and non-human recordings.

Next to material science, the thermal signature of humans has been subject to numerous scientific studies. It has been found that different medical conditions, affective state and vital parameters can be reliably analyzed with thermal imaging. The human face has been in the focus of many of these studies. Usually, a specific region of interest (ROI) in the face is analyzed. If video sequences are analyzed, then it is crucial that this ROI remains stable in order to minimize motion artifacts. In many publications, this is achieved by constraining the subject’s head motion so a fixed ROI can be used. Some recent approaches use different tracking methods to stabilize the ROI’s relative position. However, these tracking approaches are usually still rudimentary, usually tailored to the underlying problem and still require at least some manual interaction during initialization.

In our work, we therefore introduce a modular system for fully automated analysis of faces in thermal recordings. We combine several machine learning-based methods for face detection, facial landmark tracking and image analysis to form a full pipeline. Additionally, we include a face frontalization module that generates frontal views of moving faces, thereby enabling easy use of analysis methods that require frontal views. Our main contributions are:

- A set of methods for face detection and analysis. While methods for most of the addressed tasks have been published previously, we peopose combinig these approaches into a full system and also analyze the effect of different modules on the final pipeline result.
- A method for thermal face frontalization that allows straightforward transfer of available algorithms requiring frontal faces to real-world videos with unconstrained head motion.
- A thorough evaluation of the system, focusing not only on the individual modules but mainly on the interaction between these to assess wether our fully automated analysis yields any benefits or downsides compared to a partially manual workflow.

Together, all these contributions aim at providing a set of methods for improved thermal face analysis and faster development of future algorithms. Since our system provides high quality landmarks for both laboratory and real-world data, scientists can focus on developing the actual analysis algorithms and use our method for automated data preprocessing, drastically lowering the impact of head motion and acquisition conditions on the final algorithm results. This paper is an extended vetsion of [1], where we presented our system for thermal face analysis.

## 2. State of The Art

In this section, we describe existing approaches for different thermal face analysis tasks.

### 2.1. Face Detection

Often, face detection in thermal images is performed using specialized domain-specific algorithms that take advantage of the fact that certain areas of the face usually show specific temperature distributions [2] or by using thresholding methods that assume that the pixel intensity of head pixels is much higher than the background temperature [3]. While both assumptions are often true and the resulting algorithms have high computational efficiency, these methods often lack the robustness of current face detection algorithms presented for images in the visual domain. Due to their nature, they often have algorithm-specific requirements towards background structure, head pose, visible body area or camera position relative to the face. If these requirements are not met, then detection results may be highly misleading. This issue is addressed in current face detection algorithms for the visual spectrum by using machine learning techniques to reliably classify image parts containing faces with little or no restrictions towards the remaining image content. Recently, a number of authors have analyzed how these methods can be applied to thermal images as well [4,5,6]. In their research, these authors were able to show that face detectors based on machine learning constantly outperform specialized approaches if a suitable training database is provided. With increasing computing power and several recently available thermal face databases [7,8], the current trend in thermal face detection is the use of learning-based detctors.

### 2.2. Facial Landmark Detection and ROI Definition

The definition of points and regions of interest (ROIs) in faces is a crucial step in face analysis pipelines. Most methods for thermal face analysis either use fully manual ROI definitions or basic automated detection and tracking methods to detect the ROIs required for signal extraction. Manually defined ROIs or ROIs that use minimal automation are used in most current papers [9,10]. A few authors such as [11,12] use tracking methods with limited robustness to adjust their ROI positions to videos in which little facial movement is displayed. However, all authors who use these tracking methods mention that their solutions can only be applied to very limited movement ranges. The common statement made by all authors is that fixed ROIs or those that use an elementary tracking algorithm are sufficient for fully frontal data acquired under laboratory conditions where subjects are not moving, however they cannot be applied to real-word data that has been acquired under unconstrained conditions. Indeed, we believe that automated ROI definition is the key limiting factor that prohibits use of most published thermal face analysis algorithms on real-world data, therefore we will introduce methods for transforming unconstrained videos into fully frontal videos in Section 3.3.

A required step for automated ROI definition and tracking is the automated localization of salient facial regions that may serve as reference positions for ROI localization. In thermal images, many authors localize thermally well detectable regions such as eyes [13] or nostrils [12,14], however only very limited work has been published on the localization of multiple anatomic facial landmarks in thermal images. However, only a detection of a large number of landmarks yields sufficient localization information to allow ROI tracking across a large number of head poses. A step towards multi-landmark detection is [15], where different deep learning techniques are applied to detect the eyes, nose tip and mouth corners simultaneusly. A further step in this area was the publication of a massively annotated thermal face databse in [16]. The database provides 2935 high-quality thermal face images with full manual annotations of 68 landmark points, providing for the first time a dataset of comparable size and quality to current face databases in the visual domain. The database has subsequently been used to train a set of facial landmark detection methods such as feature-based Active Appearance Models [17], A Deep Alignment Network [7] and a method for deep regression of shape parameters [18]. The quality and robustness of these methods clearly surpasses previous approaches, therefore, we will focus on utilizing these for our fully automatic face processing pipeline.

### 2.3. Face Analysis

The number of publications on actual analysis methods for thermal images is vast, therefore we will cover only a number of key applications here. Survey papers such as [19,20,21] give a more in-depth overview of published applications in the medical and psychophysiological domain.

Ther perhaps most common task is breating signal analysis since the respiratory signal can be easily detected in thermal images by analyzing nostril temperature change. At the same time, this is an extremly difficult task in the visual domain. Several methods for respiratory signal extraction and analysis have been published, examples include [12] and [4]. In [22], the authors additionally used a 3D depth camera to improve tracking and detection robustness. A common feature of all presented algorithms is that they include either a manual or automatic localization of the nostrils for ROI definition and subsequently an analysis of the temperature change.

Next to respiratory rate (RR), automated heart rate (HR) extraction from thermal images is also an often addressed task. It is conceptionally similar to RR analysis since both require monitoring the temperature change in a given region, however HR analysis is more challenging due to the more subtle character of HR-induced temperature change and the higher signal frequency. Unlike respiratory signal analysis which focuses its attention on the nasal region for obvious reasons, there is also no prevailing opinion in which region the heart rate signal should be extracted. Several regions and region combinations have been analyzed in the literature [10,23,24].

A further, frequently addressed task is emotion analysis, either by analyzing facial expressions as commonly performed in the visual domain or by investigating changes in the thermal signature. The idea behind the latter is that affective changes can be detected in the thermal domain. A notable task that falls within affective state analysis is stress detection. Several authors have pointed out that mental stress can be detected by analyzing the thermal signal with [25,26,27] being notable and current publications in this area.

A third major research area is face recognition and person identification with thermal images. Notable publications include [28] and the overview given in [29].

## 3. Materials and Methods

In this chapter, we describe the components of our pipeline and the developed algorithms in detail. Additionally, we describe how these components are combined into a fully automated system for thermal face analysis. As described in the previos chapter, the tasks required for thermal face analysis are face detection, facial landmark and ROI tracking and the analysis itself, therefore we provide dedicated subsections for these tasks. All algorithms operate exclusively on thermal image data without the need for an additional visual or 3D depth camera. Wherever a machine learning algorithm was part of our algorithm, we used the annotated database introduced in [7] for training. The database contains 2935 manually annotated grayscale thermal images acquired with an InfraTec HD Head 820 thermal camera with a spatial resolution of 1024 × 768 pixels and a thermal resolution of 0.03K and using the acquisition software provided by the camera manufacturer. We trained the algorithms using the specifically marked training images of the database which includes 2356 images. Our algorithms were implemented in Python 3.6, image processing algorithms were written in scikit-image and machine learning methods were implemented with methods provided by the scikit-learn and PyTorch libraries. Note that the system we introduce in Section 3.5 is a highly modular implementation that allows changing individual components without affecting others. We take advantage of this fact by implementing and evaluating different landmark tracking methods. Therefore, new methods for either tracking or analysis can be added with minimal effort, making the system easily extensible.

### 3.1. Face Detection

As shown in [4], learning-based approaches outperform algorithm-based methods in terms of precision and especially robustness. Since we want our system to be usable in real-world conditions, only learning-based methods are an option. For thermal face detection, we trained a HOG-SVM based face detector which, according to [4], gives a good balance between runtime, robustness, precision and implementation complexity.

### 3.2. Facial Landmark Detection

The landmark detection subtask is crucial for the performance of the whole pipeline since accurately detected landmarks are a key requirement for further processing. Due to the importance of this step, we implemented and evaluated a set of different facial landmark detection algorithms.

#### 3.2.1. Active Appearance Models

Using the database, we generated a feature-based Active Appearance Model (AAM). Using the bounding box returned by the face detector and the model’s mean shape as initialization, we optimize the model parameters until optimal landmark positions are found. As shown in [17], feature-based AAMs allow precise landmark localization in thermal facial images. While yielding a high grade of precision, their main downside is the long fitting time as AAMs rely on multivariate optimization. This time can be reduced by decreasing the number of parameters and using less time-consuming feature computations, however this usually comes at the cost of reduced precision and robustness. To analyze this effect, we generated two AAMs; one parametrized for maximal precision and one with a drastically reduced parameter set for faster convergence speed. Nevertheless, AAMs should only be considered for off-line use.

#### 3.2.2. Deep Alignment Networks

To assess wether current landmark detection methods that are based on deep learning can be used in our system, we used the database to train a Deep Alignment Network (DAN) [8] for precise landmark detection. It has already been shown in [7] that DANs can be used for robust landmark detection in thermal images and yield extremly precise results. Therefore, adding this method allowed incorporating a very current and advanced method from the visual domain into our pipeline.

#### 3.2.3. Shape-Constrained Networks

Unlike AAMs, current deep learning approaches for facial landmark detection do not use prior shape information. Instead, this information is learned inherently by the networks, thereby requiring either large numbers of training samples or highly complex networks such as the DAN. Here, we use a method of combining statistical shape information with the robustness of deep convolutional networks by explicitly incorporating the statistical model into the network itself [18]. This is achieved by a principal component analysis (PCA) layer that allows generating landmark positions from PCA parameters. According to the literature, this method is faster than the previously described AAMs and DANs, yielding a precision comparable to a high-quality AAM while requiring only a fraction of the computational effort.

### 3.3. ROI Definition

**Figure 1:**
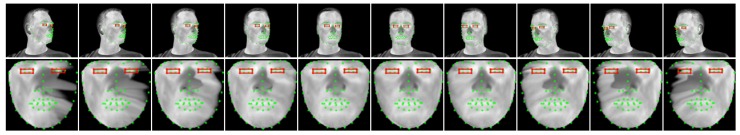
Face frontalization. Note how the ROIs move with the face in the original video feed but remain in a fixed position in the frontalized view. Severe out-of-plane rotation may distort the frontalized image, however as the image shows the amount of acceptable rotation still covers most usual head poses.

With a large number of landmarks available for every image, ROIs can be defined algorithmically by using the landmark locations and their spatial relation as reference. In addition to that, we include a method for face frontalization by piecewise affine registration of the detected facial landmarks into a canonical reference shape. In our algorithm, the face is divided into a set of triangles using delaunay triangulation of the 68 detected landmarks. Subsequently, each of the triangles is warped into a frontal reference shape using affine transformation which is computationally a highly efficient process. Our method is identical to the approach previously published in [30] to allow feature extraction from moving faces for pain detection. By using a common traingulation of both the detected facial landmarks and the reference shape, we can compute a piecewise affine transformation to each triangle and apply the set of transformations to transform a face from an arbitrary position in the image into a well-defined coordinate system. This allows using fixed ROIs for image analysis even for moving faces, thereby additionally increasing the number of algorithms that can now be applied to images with unconstrained movement. Note that the frontalization cannot replace data that is missing in the original image, i.e. if self-occlusions lead to occluded face areas, then these areas will still be transformed back into the reference frame but may contain data that is not usable for further analysis. This effect is displayed in Figure 1 where the occluded section of the face is not reconstructed properly. Note, however, that a wide range of rotations can be reconstructed with little quality loss, thereby allowing the use of analysis software even on rotated faces.

### 3.4. Analysis Modules

Our system aims at providing a platform that would allow the use of existing analysis algorithms on onconstrained data and to speed up development of novel algorithms by providing a reliable tracking backbone, allowing the user to focus on validating the analysis itself. To demonstrate and validate the viabiltiy of this approach, we implemented analysis modules that address two of the most common tasks of thermal image processing: breathing rate extraction and emotion recognition.

#### 3.4.1. Breating Rate Analysis

The breathing rate analysis is used to demonstrate how the face frontalization module can be applied to image data to use fixed ROIs on freely moving faces. After obtaining the landmarks, the face is frontalized using the method described in [30] and the average temperature of a ROI defined around the nostrils is computed.

#### 3.4.2. Emotion Recognition

The emotion recognition module is inspired by the classifier presented in [16]. We use the system to obtain a set of landmark points for the image. Subsequently, the face is cropped to the bounding box of the detected landmarks and a 10% margin is added to ensure that the outer face contour is well within the image. Finally, HOG features are extracted from the expanded bounding box and fed into a random forest classifier.

### 3.5. Combining the Components into a Modular System

In the previous sections, we have shown how each task is solved independently. In this section, we describe how the modules are connected to form a complete system for thermal face analysis.

**Figure 2:**
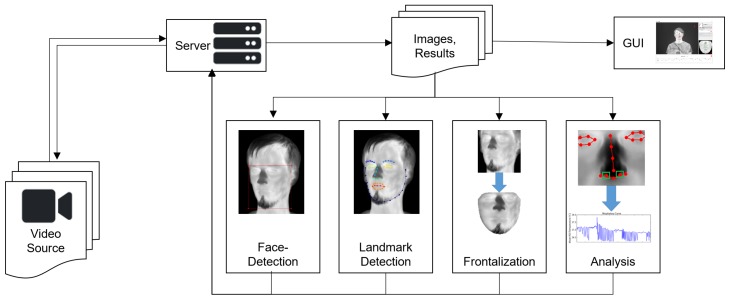
Schematic system overview: The backbone (**top row**) forwards and displays data that is processed by the specialized algorithmic modules (**bottom row**).

**Figure 3:**
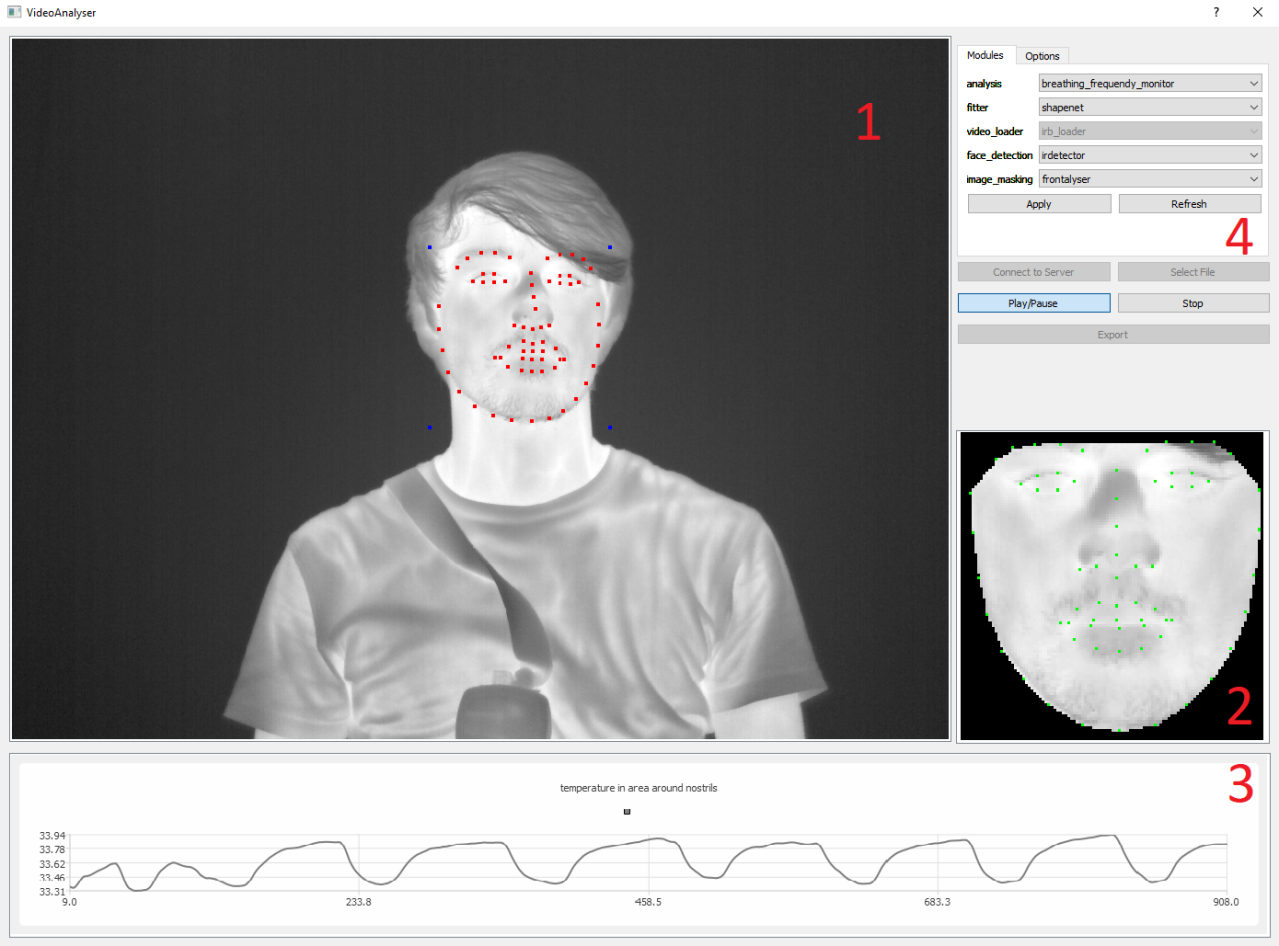
The user interface of the tracking system. 1: Live video view with overlaid landmarks from the automated ShapeNet landmark detection (red) and their respective bounding box corners (blue). 2: Frontalized view of the face for improved face analysis. 3: Output of the current analysis module, in this case a breathing rate analysis. 4: Options panel with module selection.

The backbone of the system is a ZeroMQ-based client-server system [31]. All individual parts—image acquisition and loading, face detection, facial landmark detection, frontalization and analysis—are implemented as distinct ZeroMQ nodes that receive their data from the server and send it back after processing (see Figure 2 for details). The server also forwards the data to a graphical user interface that allows selecting different modules and displaying their results. Due to the inherent robustness of the system, all modules are designed to work independently of the others. Therefore, a crashing module does not affect the entire system and modules can be interchanged at run-time, for example for switching between different trackers or analysis methods. Figure 3 shows the user interface and its components.

## 4. Experiments and Results

In this chapter, we present quantitative performance metrices for all relevant modules. Where possible, we focus on the impact of combining the individual components on the overall performance to assess if using the system yields an effect on the analysis outcome when compared to manual reference data.

Unless stated otherwise, the algorithms were trained and validated on the thermal infrared database using the training set of the database for training and videos from the test part for validation.

### 4.1. Face Detection

**Figure 4:**
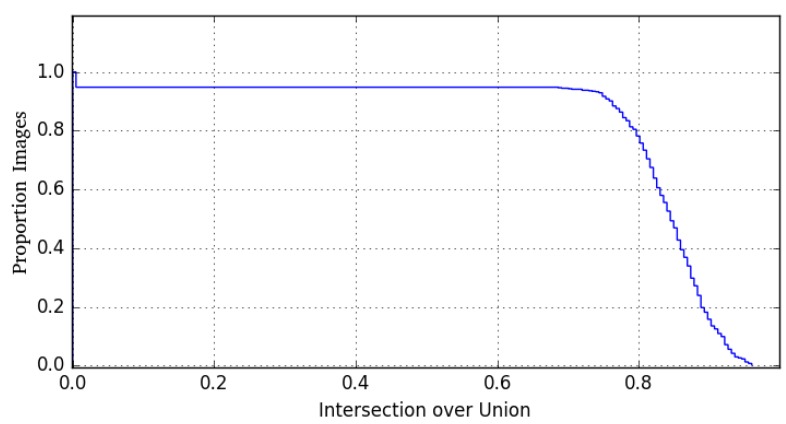
Intersection over Union (IoU) of the implemented HOG-SVM face detection algorithm. Commonly, values bayond 0.5 IoU are considered as valid and usable detections.

Figure 4 shows the Intersection over Union (IoU) of the face detection results computed by the trained HOG-SVM detector. Results show that the majority of detections is well above the threshold of 0.5 which is a commonly used value to differentiate between detections and misdetections. In fact, curve decay does not occur until an IoU of 0.7 which corresponds to a very good overall stability. However, at the same time a small number of images is not recognized at all. A closer inspection reveals that these misdetections are images with heavy out-of-plane rotation of the face. A comparison with the detection accuracy of the methods presented in [4] shows that our module yields highly comparable results.

### 4.2. Facial Landmark Detection

The landmark detection performance of the used algorithms has been extensively covered in the corresponding publications. However, all data presented so far applies to single images only. To additionally analyze the landmark detection performance on continous videos, we analyzed the results on a set of ten videos in which a total of 133 individual frames had been annotated manually. The tracking algorithms were initialized with a face detection result for the first video frame. Subsequently, tracking was performed by computing the landmark coordinates and initializing the detection of the following frame with the bounding box of the current result.

Since stable landmark detection a the key requirement for following analysis steps, we trained all previously presented detection algorithms that allow a detection of the full 68-point landmark set. Additionally, the algorithms were parametrized using different methods to analyze the impact of algorithm modifications on tracking performance:

- A high-quality AAM parametrized for maximal precision. Generated with a diagonal of 150 pixels, 36-channel DSIFT features and using the simultaneus inverse compositional (SIC) fitting algorithm, this method uses the optimal parameter set for maximal fitting precision. However, this comes at the high computational cost of about 5 s for a single frame on current consumer-grade hardware (Intel i7-6700K), correspontding to 0.2 fps and making the method only usable for offline tracking.
- A high-speed AAM using a diagonal of 70 pixels, intensity-based fitting and the project-out inverse compositional algorithm this method utilizes settings commonly used for real-time AAM fitting in the visual domain. With up to 10 fps, this method meets the runtime requirements for many real-time applications. Howevwer, the fitting performance needs to be analyzed critically since the authors of [17] have shown that these values usually result in poor fitting performance in infrared data.
- A deep alignment network trained by following the results given in [7]. With the trained algorithm, we implemented two different frame update strategies: an instance that is updated with the bounding box of the detected face (bounds-DAN) and a version that uses the detected landmark points directly for the shape update (shape-DAN).
- A ShapeNet following [18]. We trained the network with the database and also evaluated two update strategies: an instance that updates both bounding box size and position with each frame (dynamic ShapeNet) and a version that keeps a constant bounding box size and updates the face position only (fixed ShapeNet).

**Figure 5:**
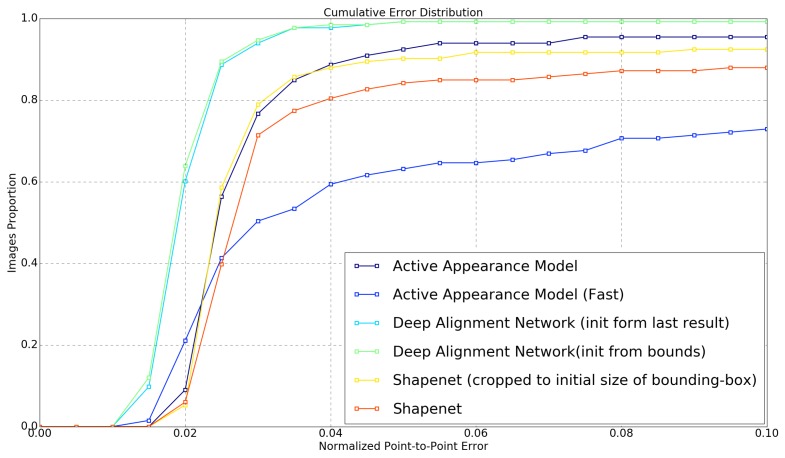
Normalized root mean squared error of the different landmark detection algorithms for quantitve performance evaluation.

**Figure 6:**
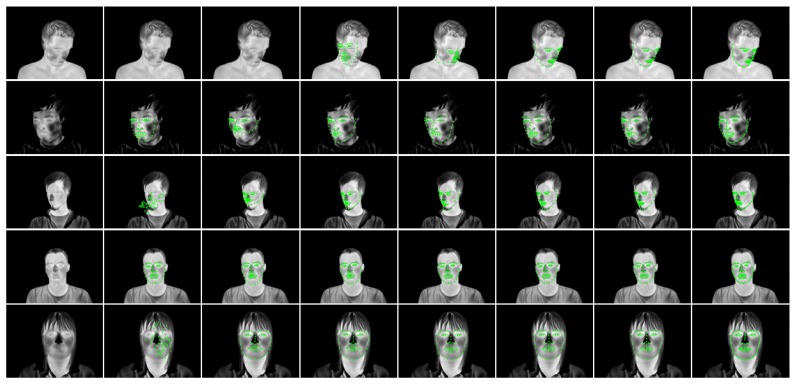
Qualitative overview of fitting performance. From left to right: Original image, fast AAM version, dynamic ShapeNet, fixed ShapeNet, high-quality AAM, boundary DAN, shape-DAN and manual ground truth. Best viewed electronically.

The results of all methods are shown in Figure 5. Both DAN methods show the best fitting accuracy, followd by the two ShapeNet implementations and the high quality AAM. The fast AAM method shows extremly low fitting precision, rendering it unusable in most cases. Note that while AAM and ShapeNet show similar fitting performances, the runtime difference is huge; ShapeNet requires about 3 ms for computing a valid result while the AAM requires 5 s per frame. A comparison of the precision of our implementation with the respective original publications shows comparable precision, although our method is able to perform fitting fully automatically on a video recording while existing literature validated precision exclusively on single images. Figure 6 shows qualitative examples of landmark detection performance. The result images qualitatively reflect the quantitative results given in the charts.

### 4.3. Emotion Recognition

**Figure 7:**
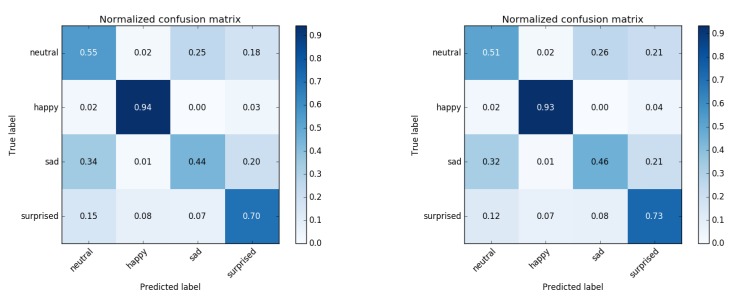
Emotion classification results. **Left**: based on the ground truth landmarks. **Right**: based on live landmarks.

We used HOG features computed from the boudning box of the detected face and trained a 1000-tree random forest classifier. For classifier training, a set of images of four basic emotions (210 neutral, 210 happy, 207 sad and 210 surprised images) were used. Although the set of basic emotions generally contains four additional emotional states (fear, anger, disgust and contempt), only four of these were explicitly included in the used database and directly usable for training. To analyze the impact of tracking on the classification result, we analyzed each image twice: first by using the manual ground truth landmarks and a second time by tracking the video containing the respective frame and performing a live classification based on landmarks detected live by the tracker. The results are shown in Figure 7. The difference between both approaches is marginal, showing that live emotion recognition using a classifier trained on still frames is well possible using our approach. With an average precision of 65.7% our implementation based on HOG features and a random forest shows a lower detection rate on the same database than the reference publication who achieved about 75% using a dense scale-invariant feature transform (DSIFT) feature extractor and a linear support vector machine (SVM) and working on the ground truth bounding boxes.

### 4.4. Respiratory Rate (RR) Analysis

**Figure 8:**
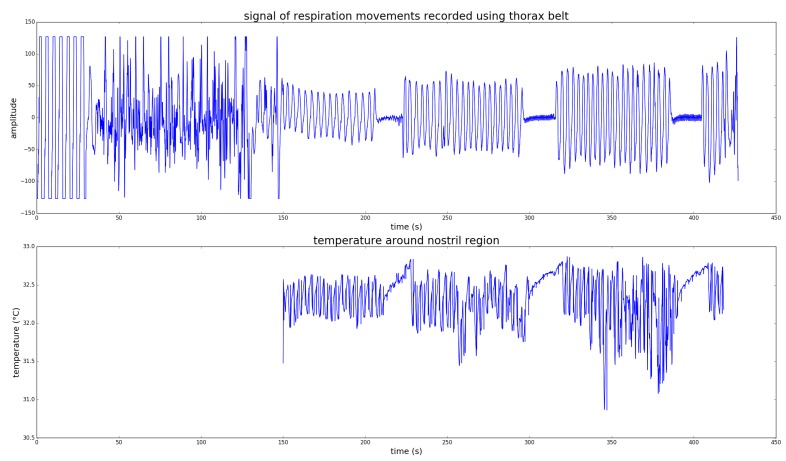
Respiratory rate extracted from the thermal signal as average nostril temperature (**bottom**) and compared to a RR signal from a commercial system (**top**).

Unlike for the other modules, RR results have only been analyzed qualitatively. The goal was to assess how well the signal extracted from the thermal images can be used for RR analysis. Figure 8 shows a representative extracted signal and the corresponding gold standard signal obtained with a commercial respiration analysis system. For the analysis, the subjects were breathing steadily for 1 min, followed by a simulated apnoea of 10–20 s. To analyze the impact of head movements, the subjects were asked to perform three sets of breathing and apnoea with increasing head motion, no motion in the first set, slow motion in the second and complex head motion in the last set. As shown in the figure, the head motion has an effect on the reconstructed RR signal, however the apnoea phases can still be identified. In this analysis, we performed no quantitative RR estimation from the respiratory signal, which is probably going to be the subject of future work.

## 5. Discussion

Our main goal was not only to implement and evaluate different algorithms for thermal face analysis, but also to develop and analyze methods for combining these efficiently into a complete system. The quantitative results show that face analysis on unconstrained tracked videos is possible. Furthermore, the proposed facial frontalization methods allow preprocessing the video data and transforming unconstrained input into frontal images, thereby enabling the use of algorithms that require frontal data. The results show that the modules can be combined without loss of precision and analysis is not impeded by using tracked data.

## 6. Conclusions

In our work, we presented a system for modular combination of several algorithms for thermal face processing. The system allows fully automatic face detection, landmark detection, face frontalization and analysis of thermal infrared face images. We were able to evaluate the performance on thermal video data and to demonstrate that using a fully automatic pipeline yields no performance loss in comparison to face analysis on manually annotated landmarks. Our future research will focus on applying the algorithms to real-world experiments involving emotion and stress detection as well as improving the robustness and precision of the presented algorithms.

We provide the software free for academic use in order to allow other researchers speeding up their research by using our algorithms to track and frontalize faces in infrared images. Contact the corresponding author for informations on obtaining the software.
